# Revisiting Cerebrospinal Fluid Flow Direction and Rate in Physiologically Based Pharmacokinetic Model

**DOI:** 10.3390/pharmaceutics14091764

**Published:** 2022-08-24

**Authors:** Makoto Hirasawa, Elizabeth C. M. de Lange

**Affiliations:** Division of Systems Pharmacology and Pharmacy, Leiden Academic Center for Drug Research, Leiden University, 2333 CC Leiden, The Netherlands

**Keywords:** cerebrospinal fluid, CSF, CSF physiology, bidirectional pulsatile CSF movement, intra-CSF administration, physiologically based pharmacokinetic model

## Abstract

The bidirectional pulsatile movement of cerebrospinal fluid (CSF), instead of the traditionally believed unidirectional and constant CSF circulation, has been demonstrated. In the present study, the structure and parameters of the CSF compartments were revisited in our comprehensive and validated central nervous system (CNS)-specific, physiologically based pharmacokinetic (PBPK) model of healthy rats (LeiCNS-PK3.0). The bidirectional and site-dependent CSF movement was incorporated into LeiCNS-PK3.0 to create the new LeiCNS-PK“3.1” model. The physiological CSF movement rates in healthy rats that are unavailable from the literature were estimated by fitting the PK data of sucrose, a CSF flow marker, after intra-CSF administration. The capability of LeiCNS-PK3.1 to describe the PK profiles of other molecules was compared with that of the original LeiCNS-PK3.0 model. LeiCNS-PK3.1 demonstrated superior description of the CSF PK profiles of a range of small molecules after intra-CSF administration over LeiCNS-PK3.0. LeiCNS-PK3.1 also retained the same level of predictability of CSF PK profiles in cisterna magna after intravenous administration. These results support the theory of bidirectional and site-dependent CSF movement across the entire CSF space over unidirectional and constant CSF circulation in healthy rats, pointing out the need to revisit the structures and parameters of CSF compartments in CNS-PBPK models.

## 1. Introduction

Cerebrospinal fluid (CSF) is a physiological medium filling the cerebral ventricles and subarachnoid space (SAS) of the brain and spinal cord. It functions to (a) protect the brain from mechanical damage by reducing its effective weight; (b) act as a drainage pathway for the removal or dilution of waste products by the “sink” effect [1,2]; (c) serve as a transport medium delivering nutrients and hormones between regions of the central nervous system (CNS) [2,3]. In addition, CSF is of great importance in the development of drug therapies for CNS diseases as the almost only accessible fluid in human CNS [4], as well being as one of the most promising administration routes for the delivery of drugs to CNS through bypassing the blood–brain barrier (BBB) [5].

However, in spite of their close contact with each other, CSF drug concentrations do not necessarily provide a reliable measure for predicting drug concentrations in brain extracellular fluid (ECF), a target site for most CNS drug candidates [4]. To overcome this limitation, CNS-specific, physiologically based pharmacokinetic (PBPK) models [6,7,8] have been developed for the prediction of brain ECF drug concentrations, utilizing available CSF concentrations. In particular, the potential of the LeiCNS-PK3.0 model [6] for predicting the unbound concentration–time profiles of small-molecule drugs in multiple physiological CNS compartments, including four CSF compartments (lateral ventricles (LV), third and fourth ventricles (TFV), cisterna magna (CM) and SAS), in rats and humans after intravenous (IV) or intramuscular administration with less than twofold error was demonstrated [6].

**Figure 1:**
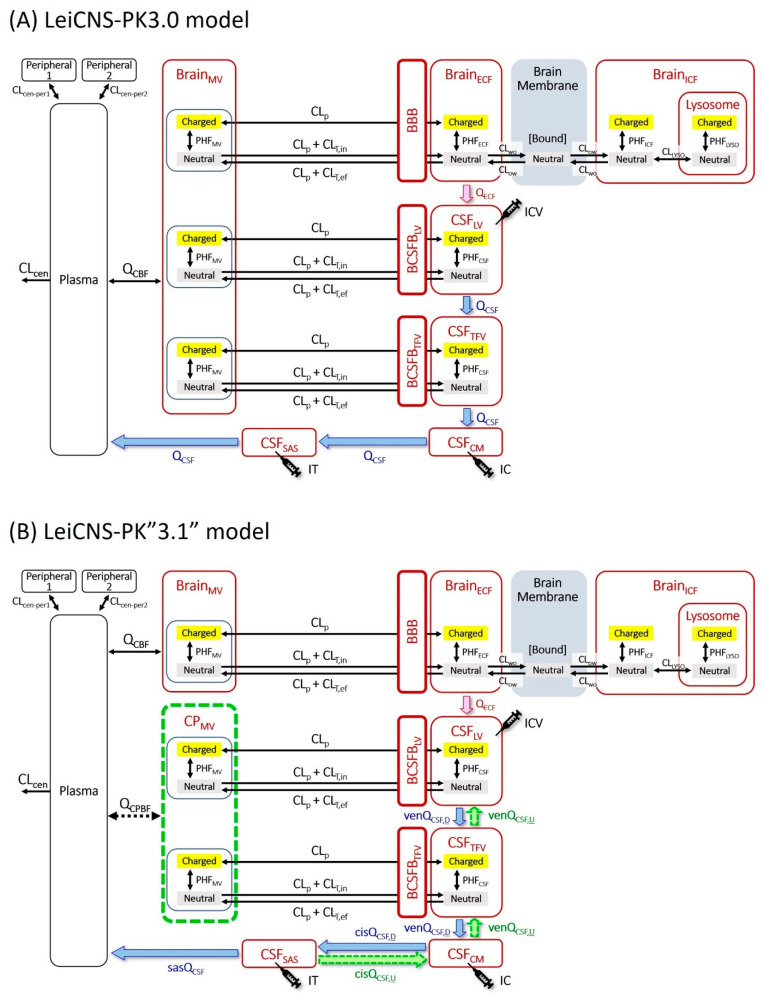
Structures of (**A**) the original LeiCNS-PK3.0 model and (**B**) the new LeiCNS-PK3.1 model. Green dashed lines represent new features of the LeiCNS-PK3.1 model. Black syringe icons represent three intra-CSF routes of administration. Barriers abbreviations: BBB—blood–brain barrier; BCSFB—blood–CSF barrier. Compartments abbreviations: CM—cisterna magna; CP—choroid plexus; CSF—cerebrospinal fluid; ECF—extracellular fluid; ICF—intracellular fluid; LV—lateral ventricles; MV—microvessel; SAS—subarachnoid space; TFV—third and fourth ventricles. Factors abbreviations: PHF—pH factor. Flow rates abbreviations: cisQ_CSF,D_—downward cisternal CSF movement rate; cisQ_CSF,U_—upward cisternal CSF movement rate; Q_CBF_—cerebral blood flow rate; Q_CPBF_—CP blood flow rate; Q_CSF_—CSF flow rate; Q_ECF_—ECF bulk flow rate; venQ_CSF,D_—downward ventricular CSF movement rate; venQ_CSF,U_—upward ventricular CSF movement rate; Clearance abbreviations: CL_cen_—central clearance; CL_cen-per1/2_—distribution clearance between central and peripheral compartments; CL_LYSO_—transmembrane clearance of lysosomes; CL_ow_—lipid-to-water clearance; CL_wo_—water-to-lipid clearance; CL_p_—paracellular transport clearance; CL_T,ef_—efflux transcellular clearance; CL_T,in_—influx transcellular clearance; sasQ_CSF_—transfer clearance from SAS to plasma; Administration routes abbreviations: IC—intracisternal; ICV—intracerebroventricular; IT—intrathecal.

CSF physiology, especially the direction and rate of CSF flow between CSF compartments, is crucially important in predicting CNS PK profiles using CNS-PBPK models, given that drug movement by diffusion is much slower than CSF flow [9]. In the LeiCNS-PK3.0 model (Figure 1A), drug movement between CSF compartments is governed by the unidirectional CSF flow from LV to TFV, CM and SAS; finally, drugs are transferred from SAS into the systemic circulation via passive CSF absorption. Constant CSF flow and absorption rates (2.2 µL/min and 0.42 mL/min in rats and humans, respectively) throughout all CSF compartments were used [6], which originate from the reported CSF-formation rates [10,11] and are based on the belief of balanced CSF formation, flow and absorption [12]. These structures and parameters in CSF compartments of the LeiCNS-PK3.0 model (and other CNS-PBPK models) are based on the traditional and classical theory of CSF physiology (known as the “third circulation” [13]), which proposes that CSF (a) is actively produced mainly by choroid plexuses (CP) in LV and TFV; (b) flows/circulates unidirectionally within the entire CSF space with a constant flow rate; (c) passively drains into venous blood mainly via arachnoid villi/granulation. This theory has been widely accepted in scientific consensus for 100 years [12,14]. However, the situation has been changing in recent years; a new concept of CSF physiology was proposed by Orešković and Klarica with a critical evaluation of the experimental methods and results on which the classical theory was based [12,14,15]. According to the new Bulat–Klarica–Orešković hypothesis: (a) there is no net CSF formation inside the brain ventricles; (b) CSF is permanently produced by water filtration across the arterial capillary walls and absorbed across the venous capillary walls in the entire CNS system depending on hydrostatic and osmotic forces; (c) CSF movement is not one of unidirectional circulation but of bidirectional (to-and-fro) oscillatory pulsation driven by heartbeat, etc.; (d) the intensity of CSF pulsation significantly varies in different CSF compartments; (e) CSF absorption into venous sinuses and/or lymphatics has minor importance due to their small surface area [12,14,15].

In order to address the need to revisit and scrutinize the structure and parameters of CSF compartments in the LeiCNS-PK3.0 model, the model’s capability to predict PK profiles in CSF compartments in healthy rats after intra-CSF (intracerebroventricular (ICV), intracisternal (IC) or intrathecal (IT; intra-lumbar CSF)) administration was checked. Next, the model’s structure and parameters were updated through adopting a theory of site-dependent, bidirectional CSF movement to more accurately describe the observed CSF PK profiles. The present study shows that a new model with bidirectional CSF movement can describe CSF PK profiles after intra-CSF administration far more accurately than the original model with unidirectional CSF flow.

## 2. Materials and Methods

### 2.1. In Vivo PK Profiles in Rats after Intra-CSF Administration

A literature search on PK data after ICV, IC or IT administration was performed using the National Library of Medicine PubMed database with search queries such as “(intraventricular [tiab] OR intracerebroventricular [tiab] OR intraventricularly [tiab] OR intracerebroventricularly [tiab] OR ICV [tiab] OR i.c.v [tiab]) AND (pharmacokinetics [tiab] OR pharmacokinetic [tiab]) AND (rats [tiab] OR rat [tiab])”. Large molecules such as proteins and nucleic acids were excluded from the dataset for the present analysis as the reported correlation between the aqueous diffusivity and molecular weight (MW), according to which the LeiCNS-PK3.0 model calculates the aqueous diffusivity of molecules, has the upper limit of MW 5000 Da [16]. In addition, the focus was on PK data in healthy rats, considering the probable disease-dependent alterations in CSF physiology in CNS disease model animals. This is also the reason that human PK data were not included in this study, which were mostly measured in patients with CNS diseases. PK data with a very short timescale (<15 min) was also excluded due to the limited information. Finally, interesting PK data of morphine after ICV administration, as reported by Bhargava et al. [17], was regrettably excluded because the continuous sampling of a large volume of CSF (>50 µL/timepoint, according to the sample volume used for radioimmunoassay; far exceeding a sum of physiological volumes of LVs, TFV and CM (32 µL) [6]) from LV of the same animal is considered very likely to impact on the normal CSF physiology. Table 1 summarizes information on PK data of ten molecules included in this study. Plasma, brain ECF and CSF PK profiles after IV administration were obtained from other literature in the case that they are not reported in the article of intra-CSF administration. Total drug concentrations in plasma were corrected using the unbound fraction in plasma. The unbound fraction in CSF was assumed to be 1 for all molecules. Kp_uu,ECF/CSF_, the ratio of the unbound drug concentration in brain ECF or CSF to that in plasma at steady state, after IV administration, was either available from the literature or calculated using the area under the unbound concentration–time curve in ECF or CSF and plasma.

### 2.2. Data Analysis and Software

WebPlotDigitizer version 4.5 [26] was used to extract data from the literature. General data analysis and visualization were performed using R version 4.0.3 [27]. Parameter estimation was performed using NONMEM version 7.4.3 [28]. Model simulations were performed using the R package “RxODE” version 0.9.2.1 [29]. Algebraic equations were solved using Maxima, a computer algebra system, version 5.44.0 [30].

### 2.3. Empirical Plasma PK Models

Plasma PK models of unbound molecules were either available from the literature or developed using plasma PK data after IV administration with a nonlinear mixed-effects modeling approach, where one-, two- or three-compartment models were evaluated. Interindividual variability (IIV) was tested for acetaminophen PK data reported by de Lange et al. [22] using exponential models for clearance (CL) and volume of distribution of the central compartment. Residual unexplained variability (RUV) was included using either proportional or combined error models. The final model was selected based on the objective function value and visual predictive check plots to compare the model fit to unbound PK profiles. Unbound plasma PK parameters used as input to the LeiCNS-PK models are summarized in Supplementary Materials Table S1.

### 2.4. Drug-Specific Physicochemical Parameters

The physicochemical parameters of ten molecules, including MW, the ionization constants of the strongest acidic group (pK_a_) and the strongest basic group (pK_b_) and lipophilicity (logP_o/w_), were collected from DrugBank version 5.1.9 [31] or the Human Metabolome Database [32] and are listed in Supplementary Materials Table S2. Experimental logP_o/w_ values were used if available, while calculated pK_a_/pK_b_ values provided by ChemAxon [33] were used.

### 2.5. Consideration of Intra-CSF Administration Volume

The physiological volumes of one LV and CM in rats (250 g of body weight) are quite small in the LeiCNS-PK3.0 model: LV—3.75 µL (reported range: 1.5–7.5 µL); CM—17 µL [6]. As shown in Table 1, in all cases, the volumes of ICV and IC dosing solutions exceed the physiological volumes of one LV and CM, respectively. It was therefore assumed that a part of the dosing solution, exceeding the physiological volume of the administration site, reaches the next compartment during administration. For example, in the case of ICV administration of 10 µL solution into the left LV, 3.75 µL of solution fills the left LV and 6.25 µL reaches the TFV at the end of administration. No upward transfer of dosing solution (TFV to contralateral LV during ICV administration and CM to TFV during IC administration) was assumed. Supplementary Materials Table S3 summarizes the assumed volumes of dosing solutions reached each compartment at the end of administration. A measure of 12.5 µL, the mid-point of other cases (10–15 µL), was assumed for ICV administration of [^3^H]sucrose, morphine and morphine-6-glucuronide, reported by Okura et al. [18], because no information on the dosing volume was reported in the article. Due to a lack of information on the duration of administration in most cases and short infusion even when this was reported (Table 1), simulations were performed under the assumption of instantaneous administration.

### 2.6. Simulation with the Original LeiCNS-PK3.0 Model

**Scheme 1:**
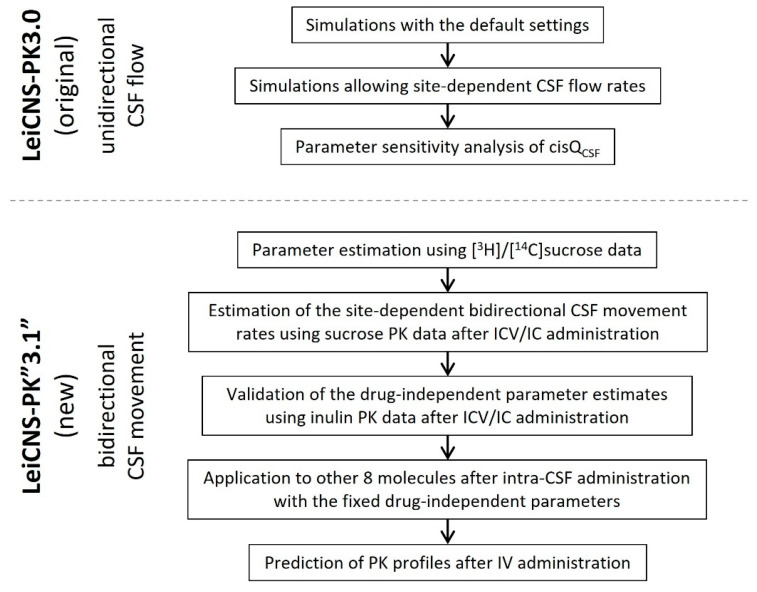
Overview of work steps in the current study with the original LeiCNS-PK3.0 model and the new LeiCNS-PK“3.1” model. cisQ_CSF_—cisternal CSF flow rate; CSF—cerebrospinal fluid; IV—intravenous.

An overview of work steps in the current study is shown in Scheme 1. Firstly, the PK profiles of ten molecules after intra-CSF administration were simulated using the LeiCNS-PK3.0 model with the default settings. Asymmetry factors (AFs), which account for active transport at the BBB or blood–CSF barrier (BCSFB) and metabolism, were calculated using Kp_uu,ECF/CSF_ values as previously reported [6]. For sucrose, neither active transport at the BBB and BCSFB nor metabolism in the CNS was assumed based on the information in the literature [34]. Similarly, the AFs of inulin were assumed to be 1.

**Figure 2:**
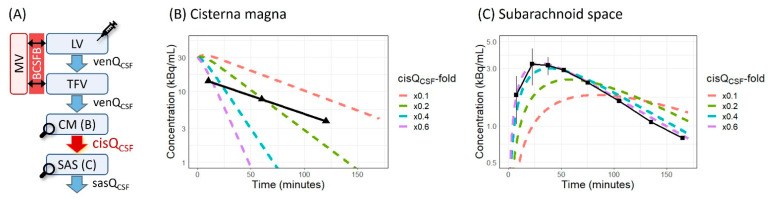
Parameter sensitivity analysis of cisternal CSF flow rate (cisQ_CSF_) in the unidirectional CSF flow model. (**A**) Model structure: cisQ_CSF_ is highlighted in red. (**B**) Simulated PK profiles in CM and (**C**) SAS. Black dots and solid lines represent mean concentrations ± standard error in CM or SAS. Dashed lines represent the median of 200 model simulations changing cisQ_CSF_ between 10% (pink) and 60% (purple) of the original Q_CSF_ value. BCSFB—blood–CSF barrier; cisQ_CSF_—cisternal CSF flow rate; CM—cisterna magna; CSF—cerebrospinal fluid; LV—lateral ventricles; MV—brain microvessel; Q_CSF_—CSF flow rate; SAS—subarachnoid space; sasQ_CSF_—transfer clearance from SAS to plasma; TFV—third and fourth ventricles; venQ_CSF_—ventricular CSF flow rate.

In order to assess the possible improvement in the description of CSF PK profiles by adopting altered CSF flow rates, further simulations were performed, allowing the site-dependent CSF flow rates. In addition, application of the correction factor to paracellular permeability (PPA) across the BBB and BCSFB was considered based on the previous finding that the LeiCNS-PK3.0 model may overestimate PPA in some cases [35]. PK data of [^3^H]sucrose reported by Okura et al. [18] was selected for these simulations for the following two reasons: (a) sucrose is known to be a CSF bulk flow marker [18], not being metabolized or bound to protein [34], which suggests that its PK profiles in CSF compartments can be considered to simply reflect the physiological CSF movement; (b) this article is the only one reporting PK profiles in multiple CSF compartments (CM and SAS) in rats after intra-CSF administration, and thus is the most appropriate to assess the impact of the site-dependent CSF flow rates on CSF PK profiles. Finally, a parameter sensitivity analysis of the CSF flow rate between CM and SAS (cisQ_CSF_ in Figure 2A), in both of which PK profile of [^3^H]sucrose is reported, was performed.

### 2.7. Introduction of the Choroid Plexus Microvessel Compartment

In the LeiCNS-PK3.0 model structure (Figure 1A), the brain microvessel (MV) compartment is directly connected to both the brain ECF (via the BBB) and CSF_LV/TFV_ (via the BCSFB) compartments. This structure allows ICV-administered molecules to traverse the BCSFB and immediately enter the brain ECF across the BBB, which is unlikely from an anatomical point of view. Simulations actually showed initial spikes in brain ECF concentrations of acetaminophen and atenolol immediately after ICV administration (Supplementary Materials Figure S1H,J), largely overestimating their brain ECF concentrations [22]. Based on this finding, a CP-specific MV (CP_MV_) compartment was separated from the whole brain MV compartment, as shown in Figure 1B. Physiological parameters on CP_MV_ were collected from the literature as follows; CP weight—4 mg/rat (250 g of body weight) [36,37]; CP blood volume—196 µL/g tissue [37]; CP blood flow rate—4 mL/min/g tissue [38] (reported range: 2.4–5.5 mL/min/g tissue [39,40]).

### 2.8. Estimation of the Site-Dependent Bidirectional CSF Movement Rates

Following the simulation results with the unidirectional CSF flow model, the bidirectional CSF movement model was considered as an alternative CSF model. Although a lot of articles reported clear evidence of the bidirectional CSF pulsation during cardiac or respiratory cycles in humans [41,42,43], dogs [44] and zebrafish [45], no information is available for rats. Therefore, the “handshake” approach [35] was adopted, by which PK data can provide information on underlying physiological parameters based on the PBPK framework. Sucrose was selected as a representative CSF flow marker for the reasons described in Section 2.6. PK data of [^3^H]sucrose in CM and SAS after ICV administration [18] and of [^14^C]sucrose in CM and plasma after IC administration [19] were simultaneously fit to the new model with the CP_MV_ compartment and the bidirectional CSF movement (Figure 1B; hereinafter referred to as the “LeiCNS-PK3.1 model”) and the following five parameters were estimated; (a) venQ_CSF,D_ (downward (craniocaudal) CSF movement rate between LV, TFV and CM), (b) cisQ_CSF,D_ (downward CSF movement rate from CM to SAS), (c) U/D ratio (the ratio between upward and downward CSF movement rates), (d) sasQ_CSF_ (transfer CL from SAS to the systemic circulation) and (e) CF_PPA_ (PPA correction factor). Assuming that the PK profiles of sucrose, as CSF bulk flow markers [18,34], reflect the physiological movement of CSF, the estimated parameters for sucrose were considered to represent the physiological CSF parameters in healthy rats. Nevertheless, it is important to note that (d) sasQ_CSF_ and (e) CF_PPA_ are likely to be drug-dependent. sasQ_CSF_ can include the transfer CL not only via passive (drug-independent) CSF absorption but also across the BBB, blood–arachnoid barrier and the blood–spinal cord barrier (BSCB) (drug-dependent) after entering parenchymal tissues by diffusion and/or via perivascular space [46,47]. Additionally, CF_PPA_ possibly accounts for the charge- and size-dependent restriction of PPA across the BBB and BCSFB, which are not accounted for in the LeiCNS-PK3.0 model [35].

Next, in order to confirm whether three drug-independent parameter estimates ((a) venQ_CSF,D_, (b) cisQ_CSF,D_ and (c) U/D ratio) derived from sucrose PK data can be used as the representative physiological CSF parameters in healthy rats, the PK data of inulin, another CSF flow marker [20], reported in two articles [40,43], were fitto the LeiCNS-PK3.1 model with three fixed drug-independent parameters, and two drug-dependent parameters were estimated.

It should be noted that the [^14^C]sucrose and [^14^C]inulin concentrations reported in the literature [19], in which no information on the counting efficiency of the detector has been reported, were converted from cpm/g to dpm/mL under the assumption that (a) CM concentrations at the end of administration are equal to the concentrations of dosing solutions; (b) densities of CSF and plasma are 1 g/mL. Given that 100 µL of the dosing solution was administered to CM after removing the same volume of CSF, CM, whose physiological volume is 17 µL [6], was considered to be filled with the dosing solution (Supplementary Materials Table S3); thus, the first assumption was reasonable.

### 2.9. Application of the LeiCNS-PK3.1 Model to Other Molecules

To further confirm the utility of the new model structure and the CSF parameters of the LeiCNS-PK3.1 model, the same analysis as the case of inulin in the previous section was performed for another eight molecules with diverse physicochemical properties (Supplementary Materials Table S2). Equations to calculate AFs were newly derived by solving the model equations at steady state in the same way as the previous report [6] and are provided in the Supplementary Equations. AFs were calculated using the derived equations and the Kp_uu,ECF/CSF_ after IV administration. In the case that Kp_uu_ < 1, efflux AFs were calculated using the relevant equation while influx AFs were set to 1, and vice versa.

### 2.10. Application to IV Administration

To evaluate the possibility of the LeiCNS-PK3.1 model as a generic CNS-PBPK model, the PK profiles after IV administration were simulated and compared to the observed data as well as the PK profiles simulated with the LeiCNS-PK3.0 model. Atenolol, acetaminophen, antipyrine, cefodizime and ziconotide were selected for this analysis because their PK profiles in plasma and CM after IV administration are reported in the same article as intra-CSF administration.

### 2.11. Further Modification of Model Structure

For morphine, acetaminophen and antipyrine, further modification of the model structure was explored to describe their observed PK profiles more accurately. Specifically, for morphine, a compartment directly connected to SAS (brain/spinal cord surface compartment (BSC) in Supplementary Materials Figure S2A), which can be interpreted to represent the CSF-facing surface of the brain, the spinal cord and possibly the arachnoid membrane, was added. A very fast exchange between SAS and BSC was assumed (Q_ex_; fixed to 100 mL/min) through considering the proximity and for parameter estimation. In addition, drug-independent transfer of CL from SAS to the systemic circulation by passive CSF absorption (sasQ_CSF,abs_) was assumed to equal to the sasQ_CSF_ of inulin, a large-molecule CSF bulk flow marker [20], and the drug-dependent CL from BSC to the systemic circulation was estimated.

For acetaminophen and antipyrine, simulations with the LeiCNS-PK3.1 model indicated that CP blood flow is the rate-limiting step in their transfer from LV/TFV to the systemic circulation due to their faster BCSFB permeability; thus, an additional CL pathway was required to more accurately describe the observed PK profiles. Therefore, a transfer CL between LV/TFV and brain MV, which can be interpreted to represent the distribution to the periventricular parenchymal tissue and subsequent elimination across the BBB, was added. Periventricular CL, as shown in Supplementary Materials Figure S3A, was estimated simultaneously with sasQ_CSF_, while CF_PPA_ was assumed to be 1.

### 2.12. Model Evaluation

Model performance was evaluated by a comparison of the predicted PK profiles with the observed ones in plasma, brain ECF and CSF, where the median and 95% prediction interval of 200 model simulations were compared to the reported unbound PK profiles. The model simulations accounted for the IIV and RUV of the plasma PK model. The relevant η of IIV and ε of RUV were randomly sampled from a normal distribution with a mean of 0 and the variance of ω^2^ and σ^2^, respectively, and transformed as required.

## 3. Results

### 3.1. Simulation with the Original LeiCNS-PK3.0 Model

As shown in Supplementary Materials Figure S1, the LeiCNS-PK3.0 model with the default settings clearly overestimated the elimination rates of seven molecules, except for acetaminophen and antipyrine, from CM after ICV administration, whereas those from SAS were predicted well in all three cases. These results indicate that the original CSF flow rate (Q_CSF_; 2.2 µL/min [6]) derived from the reported CSF-formation rates [10,48] is too fast, considering that CM concentration is determined by inflow from TFV (Q_CSF_*C_TFV_) and outflow to SAS (Q_CSF_*C_CM_), both of which are governed by Q_CSF_ in this model structure. Underprediction of initial CM concentrations of cefodizime and guanidinosuccinic acid (GSA) (Supplementary Materials Figure S1L,M) suggests that their efflux transport rates across the BCSFB in LV and TFV may also be overestimated, indicating the need to apply CF_PPA_ [35]. In addition, initial CM concentrations of IT-administered ziconotide were largely underpredicted (Supplementary Materials Figure S1N). The observed unbound concentrations of ziconotide in CM up to 60 min after IT administration were higher than those in plasma in spite of the fact that its Kp_uu,CM_ after IV administration is far less than unity (0.018), which is impossible to describe using the unidirectional CSF flow model and strongly suggests drug movement from lumbar SAS to CM against the classical CSF flow direction.

### 3.2. Parameter Sensitivity Analysis of cisQ_CSF_

Figure 2 shows the result of parameter sensitivity analysis where cisQ_CSF_ (CSF flow rate from CM to SAS; red arrow in Figure 2A) was changed from 10% to 60% of the original Q_CSF_ value (2.2 µL/min) with other parameters fixed (but modified from the default settings). The simulated PK profiles of [^3^H]sucrose in CM and SAS were compared to the observed ones. At 60%, the elimination rate of [^3^H]sucrose from CM was largely overpredicted (Figure 2B), while its mean PK profile in SAS was perfectly captured (Figure 2C). In contrast, at 10%, the elimination rate from CM was well described, while it was too slow to describe its overall PK profile in SAS. These results indicate that the unidirectional CSF flow model structure is unable to simultaneously describe the PK profiles of [^3^H]sucrose in CM and SAS, especially similar elimination rates from these compartments, even though the site-dependent CSF flow rates were applied.

### 3.3. Estimation of the Site-Dependent Bidirectional CSF Movement Rates

**Figure 3:**
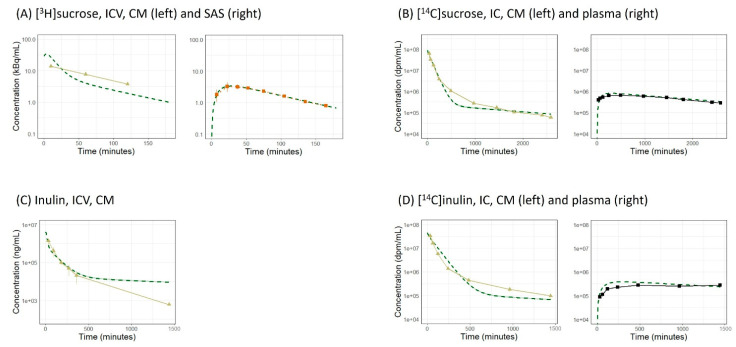
Comparison of simulated PK profiles of two CSF bulk flow marker molecules with the LeiCNS-PK3.1 model to the observed ones after intra-CSF administration. Dots and solid lines represent mean concentrations ± standard deviation or standard error in CM (khaki), SAS (orange) and plasma (black). Dashed lines represent the median of 200 model simulations with 95% prediction intervals (colored band). (**A**) [^3^H]sucrose after ICV administration, (**B**) [^14^C]sucrose after IC administration, (**C**) inulin after ICV administration, and (**D**) [^14^C]inulin after IC administration. CM—cisterna magna; CSF—cerebrospinal fluid; IC—intracisternal; ICV—intracerebroventricular; LV—lateral ventricles; SAS—subarachnoid space.

Table 2 summarizes the parameter estimates obtained by the “handshake” approach [35], i.e., fitting the observed PK data to the LeiCNS-PK3.1 model. Based on the PK data of sucrose, a CSF flow marker, cisternal CSF movement (cisQ_CSF,D_), was estimated to be faster than ventricular CSF movement (venQ_CSF,D_). Interestingly, the U/D ratio was estimated to be higher than 1, indicating an upward (caudocranial) net CSF flow. As shown in Figure 3A,B, the PK profiles of [^3^H]sucrose in CM and SAS after ICV administration were quite reasonably described by the LeiCNS-PK3.1 model, especially their similar elimination rates, which were impossible to describe with the unidirectional CSF flow model, and those of [^14^C]sucrose in CM and plasma after IC administration. Furthermore, when three parameter values estimated using sucrose data were fixed as the drug-independent physiological CSF parameters, the PK profiles of inulin, another CSF flow marker molecule [20] in CM and plasma after ICV and IC administration was also well described (Figure 3C,D) in combination with the two estimated drug-dependent parameters. A relatively large deviation between the predicted and observed CM PK profiles of inulin at later timepoints after ICV administration (Figure 3C) can simply be attributed to the plasma PK parameters, given that CM concentrations at the elimination phase are dependent on plasma concentrations, not on the CSF parameters. Although the plasma PK parameters derived from another article [19] were used in this case because no plasma PK profiles after IV administration were reported in the original article [20] (Table 1), the use of PK parameters reported in the same article further improved the description of the overall PK profile in plasma.

### 3.4. Application of the LeiCNS-PK3.1 Model to Other Molecules

**Figure 4:**
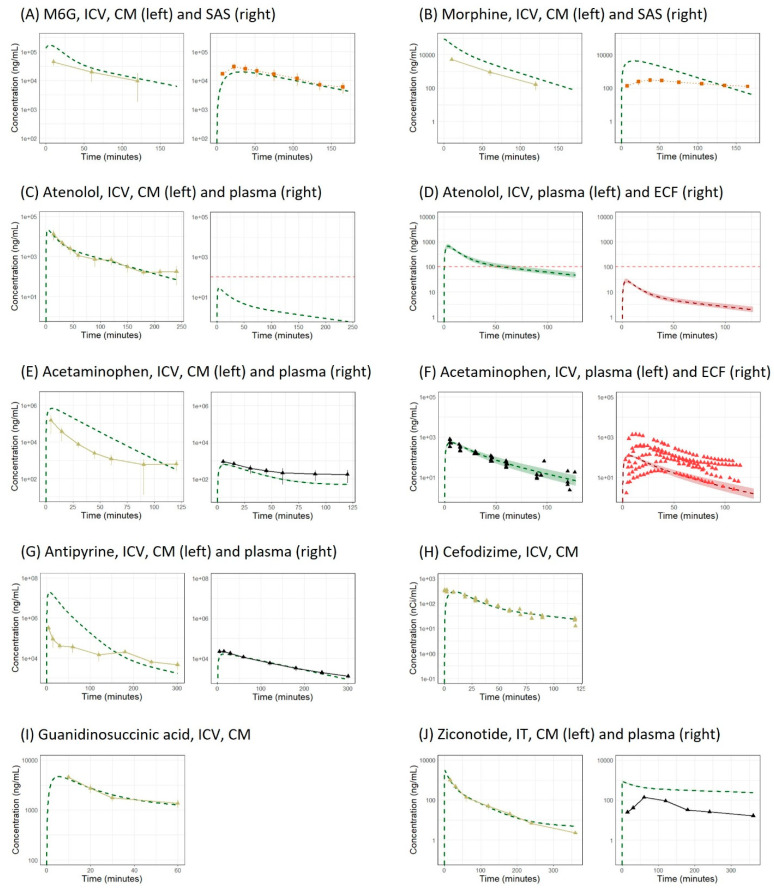
Comparison of simulated PK profiles of eight molecules with the LeiCNS-PK3.1 model to the observed ones after intra-CSF administration. Dots and solid lines represent mean concentrations ± standard deviation or standard error in CM (khaki), SAS (orange), plasma (black) and ECF (red). Dashed lines represent the median of 200 model simulations with 95% prediction intervals (colored band). Red horizontal dashed lines in (**C**,**D**) represent the lower limit of quantification of atenolol. (**A**) morphine-6-glucuronide after ICV administration, (**B**) morphine after ICV administration, (**C**,**D**) atenolol after ICV administration, (**E**,**F**) acetaminophen after ICV administration, (**G**) antipyrine after ICV administration, (**H**) cefodizime after ICV administration, (**I**) guanidinosuccinic acid after ICV administration and (**J**) ziconotide after IT administration. CM—cisterna magna; ECF—brain extracellular fluid; ICV—intracerebroventricular; IT—intrathecal; M6G—morphine-6-glucuronide; SAS—subarachnoid space.

As shown in Figure 4, the LeiCNS-PK3.1 model captured the PK profiles of eight molecules well, with the exception of morphine in SAS (Figure 4B), acetaminophen in CM (Figure 4E), antipyrine in CM (Figure 4G) and ziconotide in plasma (Figure 4J), with the combination of three fixed drug-independent parameters estimated using sucrose data and two estimated drug-dependent parameters (Table 2). Plasma concentrations of atenolol were also overestimated (Figure 4D), which were reported to be below 100 ng/mL at all time points [22]. Adopting the CP_MV_ compartment removed irrelevant initial spikes in brain ECF concentrations of acetaminophen and atenolol immediately after ICV administration (Figure 4D,F). As shown in Table 2, estimated CF_PPA_ was less than 1 except for morphine, acetaminophen and antipyrine, for which the CSF PK profile was relatively poorly described, which is consistent with the previous finding of PPA overestimation by the LeiCNS-PK3.0 model [35]. As expected, sasQ_CSF_ was estimated to be drug-dependent and inulin showed the lowest value, with the exception of GSA, which showed a negative value.

### 3.5. Application to IV Administration

**Figure 5:**
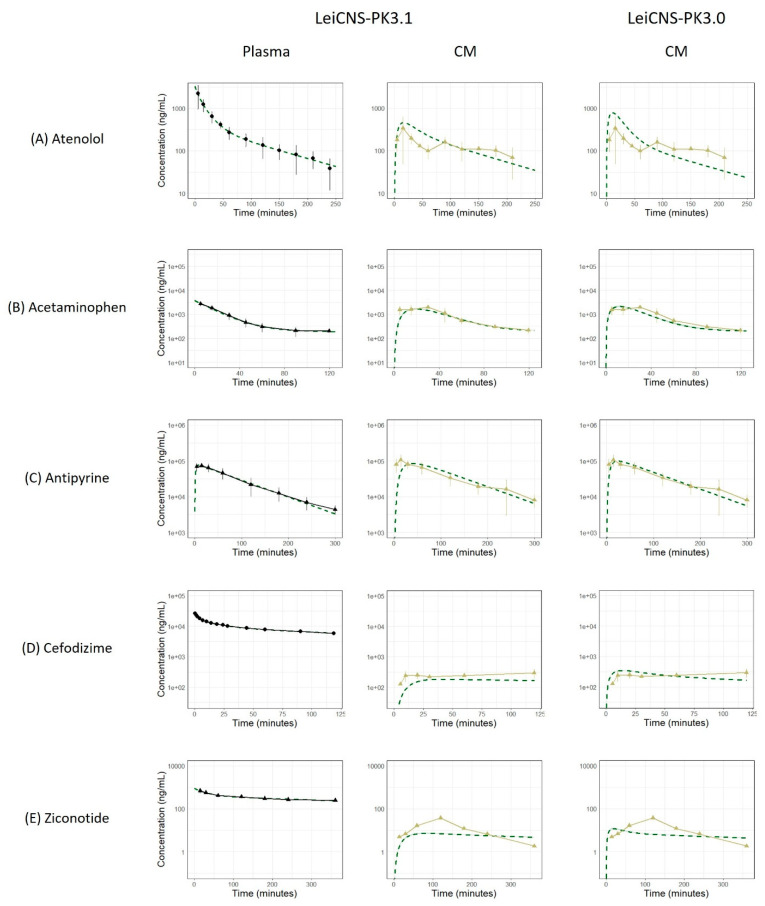
Comparison of PK profiles after IV administration in plasma (left) and CM (middle) simulated with the LeiCNS-PK3.1 model to those in CM (right) simulated with the LeiCNS-PK3.0 model. Dots and solid lines represent mean concentrations ± standard deviation or standard error in CM (khaki) and plasma (black). Dashed lines represent the median of 200 model simulations with 95% prediction intervals (colored band). (**A**) Atenolol, (**B**) acetaminophen, (**C**) antipyrine, (**D**) cefodizime and (**E**) ziconotide. CM—cisterna magna.

Plasma and CM PK profiles of atenolol, acetaminophen, antipyrine, cefodizime and ziconotide after IV administration were simulated with the LeiCNS-PK3.1 model using parameters in Table 2, as shown in Figure 5, with a comparison to those simulated with the original LeiCNS-PK3.0 model. The LeiCNS-PK3.1 model demonstrated the same level of predictability of the CM PK profiles after IV administration as the original model for all five molecules.

### 3.6. Further Modification of Model Structure

For morphine, the addition of BSC allowed us to describe the SAS PK profile after ICV administration far more accurately (Supplementary Materials Figure S2D). The estimated distribution volume of BSC (5.24 mL) was much larger than the physiological volume of the whole brain (1.8 mL), which may indicate the strong binding of morphine to CSF-facing parenchymal tissues.

Similarly, the addition of periventricular CL clearly improved the description of the CM PK profiles of acetaminophen and antipyrine (Supplementary Materials Figure S3) after ICV administration, possibly indicating their distribution to the periventricular parenchymal tissue and subsequent elimination across the BBB. These results indicate that the original Q_CSF_ in the LeiCNS-PK3.0 model were fast enough to describe the CM PK profiles of these two drugs relatively well (Supplementary Materials Figure S1I,K).

## 4. Discussion

Given the clearly demonstrated bidirectional pulsatile CSF movement in humans and the new concept of CSF physiology, the structures and parameters of CSF compartments in CNS-PBPK models need to be revisited. In the present study, the capability of the new LeiCNS-PK3.1 model with the adapted bidirectional and site-dependent CSF movement to describe the reported PK data of small molecules after intra-CSF administration in healthy rats was compared with that of the original LeiCNS-PK3.0 model with unidirectional and constant CSF flow. The present study shows that our new model can describe CSF PK profiles after intra-CSF administration far more accurately than the original model.

### 4.1. CSF Compartments and Parameters in CNS-Specific PBPK Models

Several kinds of CNS-specific PBPK models have been reported and have demonstrated their utility to describe PK profiles in multiple CNS compartments [6,7,8,49,50,51]. Although they have different model structures, e.g., the number of CSF compartments, the use of CSF flow and/or absorption rates originating from the CSF-formation rate at the CP, based on the classical theory of CSF physiology, is common among them. The 4Brain model [8] implemented in the Simcyp Simulator adopts the bidirectional “shuttle” CSF flows between cranial and spinal CSF compartments, but their rates are calculated considering the net CSF absorption from the spinal CSF compartment and are still based on the traditional CSF-formation rate.

According to Orešković and Klarica, however, the experimental methods used to determine CSF-formation rate have been insufficiently scientifically evaluated and the results are of questionable reliability; moreover, a direct determination of CSF-formation rate suggests that there is no net CSF formation inside the brain ventricles [12,15]. Instead, they proposed that CSF and ECF are created by water filtration through the arterial capillary walls across the entire CNS. These critical views and new concepts of CSF formation imply that sticking to the traditional CSF-formation rate as a basis may lead to misinterpretation of CSF movement rate, which governs substance movements in CSF compartments, and are therefore worth taking into account.

Given this scientific context, the structure and parameters of CSF compartments in our CNS-PBPK model were revisited. Our approach to exploring the physiologically relevant CSF model here was to utilize the existing PK data under the assumption that the PK profiles of known CSF flow marker molecules simply reflect physiological CSF movement. A recent article by Monine et al. [52] demonstrated that their CNS-PBPK model with bidirectional mass transfer flows between CSF compartments can describe the distribution of IT-administered antisense oligonucleotides (ASOs) in nonhuman primates. However, they focused on ASOs, and it is unclear whether the estimated transfer rate constants for ASOs relevantly reflect the physiological CSF movement. Furthermore, the estimation of transfer flows between CSF compartments faced a problem of unidentifiability, partly because the CSF concentrations included in the analysis were only of lumbar CSF, while ASO concentrations in parenchymal tissues were measured in multiple regions. Accordingly, as far as we know, the present paper is the first publication reporting a possibly physiological, bidirectional CSF movement rate in healthy animals based on PK data.

In addition, this is surprisingly the first modeling study analyzing CSF PK data of small molecules after intra-CSF administration using a CNS-PBPK model, even though a relevant model should be able to describe PK profiles after intra-CSF administration as well as systemic administration [5].

### 4.2. Simulation by the Original LeiCNS-PK3.0 Model with the Unidirectional CSF Flow

Chang et al. [50] demonstrated that CSF PK profiles of ICV- or IT-administered antibodies can be captured well by their CNS-PBPK model with a unidirectional CSF flow, whose rate originates from the traditional CSF-formation rate, plus the glymphatic flow from SAS to ECF. Nevertheless, as shown in Supplementary Materials Figure S1, in this study, the PK profiles in the CM and SAS of most molecules after intra-CSF administration were difficult to describe by the LeiCNS-PK3.0 model, with very similar structures and parameters of CSF compartments to those used in Chang’s model except for glymphatic flow.

Further analysis revealed that it is impossible to simultaneously describe PK profiles of [^3^H]sucrose in CM and SAS after ICV administration with a unidirectional CSF flow model, even allowing for the site-dependent CSF flow rates (Figure 2). These results motivated an exploration of other possible model structures of CSF compartments that can accurately describe sucrose PK profiles. Altered model structures with unidirectional CSF flow, such as multiple SAS compartments and diverged CSF flow at CM, were also considered, but it was concluded that they are unlikely to improve the description of sucrose PK profiles.

### 4.3. Bidirectional and Pulsatile CSF Movement

The bidirectional and pulsatile movement of CSF in humans was already reported in 1966 [53]; since then, it has been further demonstrated by many studies by means of phase-contrast magnetic resonance imaging [41,42,43,54,55,56,57] as well as through the use of the time–spatial spin-labeling inversion-pulse method [58]. These imaging techniques are noninvasive and have enabled investigation of further details of the pulsatile CSF movement in humans, such as the correlation between CSF pulsation amplitudes and changes in cerebral blood stroke volume [54,55], a larger impact of respiration as the driving force [43], age- and sex-dependent implications [42], and disease-related alterations [41,56,57,58]. Accordingly, the existence of the bidirectional pulsatile CSF movement, instead of a constantly unidirectional flow, is an undoubted fact at least in human, dog [44] and zebrafish [45], and it should be considered as a physiologically relevant process.

Importantly, bidirectional pulsatile CSF movement allows substances in the CSF to mix and spread in both directions. This is clearly demonstrated in the study reported by Smith et al. [59], where ^111^In- and ^99m^Tc-labeled diethylenetriamine pentaacetate (DTPA) were administered to the right LV and lumbar CSF, respectively, of the same patient at almost the same time, and time-dependent distribution from the injection site to the other site was observed in both directions. [^99m^Tc]DTPA concentrations in the right LV after IT administration cannot be explained by its re-entry to CSF across the BCSFB after first being absorbed into systemic circulation given the poor BCSFB penetration of DTPA-chelates [60]; thus, its distribution from lumbar SAS to the LV is a result of the upward movement of [^99m^Tc]DTPA against the traditional CSF flow direction.

### 4.4. Parameter Estimates Possibly Representing the Physiological CSF Values in Healthy Rats

In this study, the new LeiCNS-PK3.1 model with a bidirectional and site-dependent CSF movement demonstrated the superior description of PK profiles of [^3^H]sucrose in CM and SAS after ICV administration, especially their similar elimination rates, as well as that of [^14^C]sucrose in CM after IC administration, in comparison with the original LeiCNS-PK3.0 model, which had unidirectional and constant CSF flow (Figure 3A,B). More importantly, this model, with three fixed drug-independent parameters estimated using sucrose PK data, was able to describe the CM PK profiles of most of the molecules tested, including another CSF flow marker, inulin, after intra-CSF administration (Figure 3 and Figure 4). These results suggest that these parameter estimates can possibly be used as representative values of physiological CSF parameters in healthy rats. Nevertheless, to this end, these parameter values must be supported by other physiological findings.

First, the movement rate of CSF from CM to SAS (cisQ_CSF,D_) was estimated to be four times higher than that from LV to TFV and from TFV to CM (venQ_CSF,D_) (Table 2). This order is consistent with clinical observations, where CSF volumetric net movement per cardiac cycle or during forced breathing was much lower at the cerebral aqueduct than at the cranio–cervical junction (CCJ) [41] or the upper thoracic spinal canal [43]. This could also make intuitive sense given the narrower radius of the aqueduct having higher resistance to CSF movement.

Second, the U/D ratio was estimated to be higher than 1, indicating an upward (caudocranial) net CSF flow throughout all CSF compartments. Although this is difficult to intuitively understand from the viewpoint of the classical CSF physiology, some clinical results support this finding. Reported U/D ratio of CSF movement per heartbeat at the cerebral aqueduct was around 1.4 [42], close to the estimate in rats in this study. In addition, CSF net flow per forced breathing cycle was in an upward direction at the aqueduct and thoracic level 2 in almost all subjects [43]. It is also worth noting that upward transfer of ASOs between CSF compartments in nonhuman primates was estimated to be faster than the downward transfer [52]. However, it should be noted that there are contradictory observations, and the net CSF direction is still questionable [15]. For example, Eide et al. reported that the CSF volumetric net flow per cardiac cycle at the cerebral aqueduct was downward in 69% of human subjects, whereas they reported that, at CCJ, the volumetric net flow was upward in 78% [41]. Normal net CSF flow at the aqueduct in beagle dogs was downward in most cases [44]. On the other hand, the time–spatial spin-labeling inversion-pulse method for labeling CSF itself demonstrated that there is no dominant net CSF flow in humans because systolic and diastolic CSF movements are equal [15,58]. Furthermore, CSF volume redistribution can occur during body position changes and other activities [15]. Taken together, the net CSF flow direction could be condition-dependent and possibly even species-dependent. Given these findings, however, the successful description of PK profiles of most molecules in healthy rats with the LeiCNS-PK3.1 model, in spite of the fact that they were reported from multiple laboratories with different experimental conditions, should be considered meaningful.

### 4.5. Possibility of the LeiCNS-PK3.1 Model as a Generic CNS-PBPK Model

Importantly, the LeiCNS-PK3.1 model showed the same level of predictability of CM PK profiles after IV administration as the original LeiCNS-PK3.0 model (Figure 5). Given that the LeiCNS-PK3.0 model has been validated as a generic CNS-PBPK model for systemic administration not only in rats but also in humans [6], it is expected that the LeiCNS-PK3.1 model can be used as a more generic CNS-PBPK model, applicable to intra-CSF as well as systemic administration.

### 4.6. Limitations

Nevertheless, there are limitations and certain points remain to be improved in the LeiCNS-PK3.1 model. First, the parameter values we consider as representing the physiological CSF parameters in healthy rats completely depended on PK data of radioisotope-labeled sucrose, as reported in two articles [18,19]. In particular, the PK data of [^3^H]sucrose in lumbar CSF after ICV administration reported by Okura et al. [18] was determined by the microdialysis method, which might sometimes cause tissue damage [4]. Although we believe that the overall results of this study strongly support the validity of these values for healthy rats, it should be noted that the current dataset might include PK data in non-physiological conditions. With more data available to validate and possibly revise these parameters, confidence in the model structure could be increased.

Additionally, in this analysis, the impact of the dosing speed on CSF PK profiles was not considered and the volume of the dosing solution reached each compartment at the end of administration was simply assumed. Computational fluid dynamics analysis [61] based on the Navier–Stokes equation will allow appropriate descriptions of the impacts of dosing speed and administration-derived convective flow.

Next, as shown in Supplementary Materials Figures S2 and S3, further modifications to the model structure clearly improved the description of the PK profiles of morphine, acetaminophen and antipyrine, which indicates the need to incorporate the distribution process to SAS-facing and periventricular parenchymal tissues. The structure and parameters of these exploratory models are not physiologically based at this moment, and thus need to be updated further. To this end, more in vivo data at multiple CSF sites and about molecules with more diverse physicochemical properties are required. Overestimation of the plasma exposure of atenolol after ICV administration (Figure 4D) may also be solved by incorporating periventricular distribution. On the other hand, overestimation of the plasma exposure of ziconotide after IT administration (Figure 4J) occurs due to its low systemic bioavailability [25], through a mechanism that remains unclear; it was possible to better capture this by applying the bioavailability calculated from the reported area under the concentration–time curve (data not shown). This case suggests that metabolism or degradation in CSF, as well as possibly strong binding or adsorption to CNS components, also need to be considered for some cases.

In addition, large interindividual variability in the ECF concentrations of acetaminophen after ICV administration (Figure 4F) [22] can be attributed to the differences in spatial positions of microdialysis probes, as confirmed in [62]. Three-dimensional modeling approaches [63] can allow the precise description of such local PK profiles.

Furthermore, overestimation of PPA based on molecular aqueous diffusivity was indicated for the BCSFB, as with the previous study for the BBB [35]. This is noted especially for highly hydrophilic molecules because of size- and charge-restricted paracellular pores, which need to be accounted for in future models.

As far as we know, this is the first modeling study using a CNS-PBPK model to show the drug-dependent transfer CL from SAS to the systemic circulation (sasQ_CSF_), which is treated as a drug-independent passive CL in other CNS-PBPK models, with the exception of applying the lymph reflection coefficient for antibodies [50]. This is quite reasonable in light of recent extensive research on the glymphatic system or the exchange between the CSF and ECF of the CSF-facing parenchyma of the brain and spinal cord via the perivascular space [46,64,65,66], followed by the drug-dependent transport of CL across the BBB and BSCB. However, the relative contribution of each pathway (including the glymphatic system, the lymphatic pathway, the vascular pathway via the BBB/BSCB and arachnoid granulation) to the overall process of waste CL from CNS is yet to be quantitatively established [15,67]. Hence, sasQ_CSF_ is currently impossible to predict from physicochemical properties, in vitro transporter studies or in vivo Kp_uu,ECF/CSF_ values, and can be estimated only with in vivo PK profiles and model fitting.

When estimating using in vivo data, the selection of timepoints is important. For example, the sasQ_CSF_ of GSA was estimated to be below 0 (Table 2), indicating a net influx from the systemic circulation directly to SAS. It is possible that this truly represents the active influx transport at the blood–arachnoid barrier, given the fact that GSA is a substrate of some solute carrier transporters [24]. Nevertheless, careful consideration of the fact that this estimate is actually dependent on data at 60 min is required; additionally, it is important to note that its CM PK profile up to 30 min can be described well even with a positive sasQ_CSF_ value. Accordingly, additional datapoints are necessary to estimate this value with assurance.

This study pointed out the importance of considering the bidirectional pulsatile CSF movement whose rate is not necessarily balanced with the traditional CSF-formation rate, instead of classical unidirectional and constant CSF flow, in the CSF compartments of CNS-PBPK models. Given the relatively rich data on pulsatile CSF movement and imaging data with various contrast agents, most of which are considered to have characteristics as CSF flow markers, in clinical situations, similar analyses of human CSF compartments will be a significant and meaningful subject of future research.

## 5. Conclusions

The LeiCNS-PK3.1 model, with bidirectional and site-dependent CSF movement, demonstrated superior performance in describing the PK profiles of small molecules after intra-CSF administration in comparison with the original LeiCNS-PK3.0 model, with unidirectional and constant CSF flow. Given the clearly demonstrated bidirectional movement of CSF in humans and the new concept of CSF physiology, the structures and parameters of the CSF compartments in CNS-PBPK models need to be revisited.

## Figures and Tables

**Table 1:** Summary of in vivo PK data after intra-CSF and IV administration included in this study.

Molecule	Reference	Dosing Route	Dosing Volume (µL)	Dosing Duration (s)	Dose	Reported PK Profile			
						CM	SAS	ECF	PL
[^3^H]sucrose	[18]	ICV	12.5 ^a^	NR	5 kBq	√	√	-	-
[^14^C]sucrose	[19]	IC	100 ^b^	NR	4 µCi	√	-	-	√
		IV	100	NR	4 µCi	√	-	-	√
Inulin	[20]	ICV	12.5 ^c^	NR	2.5 mg/kg	√	-	-	-
[^14^C]inulin	[19]	IC	100 ^b^	NR	2 µCi	√	-	-	√
		IV	100	NR	2 µCi	√	-	-	√
Morphine-6-glucuronide	[18]	ICV	12.5 ^a^	NR	50 nmol	√	√	-	√ ^d^
Morphine	[18]	ICV	12.5 ^a^	NR	50 nmol	√	√	-	√ ^e^
Atenolol	[21]	ICV	10	5–10	12 µg	√	-	-	√ ^d^
		IV	500	60	1 mg	√	-	-	√
	[22]	ICV	15	5	150 µg	√	-	√ ^d^	√ ^d^
		IV	500	60	10 mg	√	-	√	√
Acetaminophen	[21]	ICV	10	5–10	210 µg	√	-	-	√
		IV	500	60	825 µg	√	-	-	√
	[22]	ICV	10	5	210 µg	√	-	√	√
		IV	500	60	825 µg	√	-	√	√
Antipyrine	[21]	ICV	10	5–10	5 mg	√	-	-	√
		IV	500	60	20 mg	√	-	-	√
Cefodizime	[23]	ICV	10	NR	0.074 µCi	√	-	-	-
		IV	1 ^f^	NR	100 µCi/kg	√	-	√	√
Guanidinosuccinic acid	[24]	ICV	10	NR	0.05 µmol/kg	√	-	-	-
		IV	NR	NR	1 µmol/kg	√	-	-	√
Ziconotide	[25]	IT	100	NR	100 µg/kg	√	-	-	√
		IV	100	NR	100 µg/kg	√	-	-	√

CM—cisterna manga; ECF—brain extracellular fluid; IC—intracisternal; ICV—intracerebroventricular; IT—intrathecal; IV—intravenous; NR—not reported; PL—plasma; SAS—subarachnoid space. ^a^ Assumed as the middle of other ICV cases (10–15 µL); ^b^ the same volume of CSF was withdrawn before administration; ^c^ 50 µL/kg in the literature; ^d^ below the limit of quantification; ^e^ one point only; ^f^ mL/kg.

**Table 2:** Parameter estimates obtained by fitting PK data to the LeiCNS-PK3.1 model.

Molecule	Reference	Dosing Route	Parameter Estimates				
			venQ_CSF,D_ (µL/min)	cisQ_CSF,D_ (µL/min)	U/D Ratio	sasQ_CSF_ (µL/min)	CF_PPA_
[^3^H]sucrose	[18]	ICV	0.251	1.07	1.59	1.41	0.0387
[^14^C]sucrose	[19]	IC					
Inulin	[20]	ICV	- ^a^	- ^a^	- ^a^	1.22	0.262
[^14^C]inulin	[19]	IC					
Morphine-6-glucuronide	[18]	ICV	- ^a^	- ^a^	- ^a^	1.52	0.00268
Morphine	[18]	ICV	- ^a^	- ^a^	- ^a^	4.75 ^b^	95.2 ^b^
Atenolol	[21]	ICV	- ^a^	- ^a^	- ^a^	2.22	0.685
Acetaminophen	[21]	ICV	- ^a^	- ^a^	- ^a^	15.0 ^c^	529 ^c^
Antipyrine	[21]	ICV	- ^a^	- ^a^	- ^a^	7.18 ^c^	48.0 ^c^
Cefodizime	[23]	ICV	- ^a^	- ^a^	- ^a^	1.38	0.00308
Guanidinosuccinic acid	[24]	ICV	- ^a^	- ^a^	- ^a^	−0.411	0.000239
Ziconotide	[25]	IT	- ^a^	- ^a^	- ^a^	763	0.00121

CF_PPA_—correction factor for paracellular permeability; cisQ_CSF,D_—downward CSF movement rate from CM to SAS; CM—cisterna manga; CSF—cerebrospinal fluid; IC—intracisternal; ICV—intracerebroventricular; IT—intrathecal; SAS—subarachnoid space; sasQ_CSF_—transfer CL from SAS to the systemic circulation; U/D ratio—the ratio between upward (caudocranial) and downward CSF movement rates; venQ_CSF,D_—downward (craniocaudal) CSF movement rate between LV, TFV and CM. ^a^ Fixed to values estimated using sucrose data; ^b^ poor description of SAS PK profile; ^c^ poor description of CM PK profile.

## Data Availability

The data presented in this study are available on request from the corresponding author. The data are not publicly available due to privacy.

## Supplementary Materials

The following supporting information can be downloaded at: https://www.mdpi.com/article/10.3390/pharmaceutics14091764/s1, Supplementary Equations, Table S1: Unbound plasma PK parameters used as input to the LeiCNS-PK models, Table S2: Drug-specific physicochemical parameters, unbound fraction in plasma and Kpuu values, Table S3: Assumed volumes of dosing solution reached each compartment at the end of administration, Figure S1: Comparison of simulated PK profiles of 10 molecules using the original LeiCNS-PK3.0 model with the default settings to the observed ones after intra-CSF administration. Dots and solid lines represent mean concentrations ± standard deviation or standard error in CM (khaki), SAS (orange), plasma (black) and ECF (red). Dashed lines represent the median of 200 model simulations with 95% prediction intervals (colored band). Red horizontal dashed lines in (G) and (H) represent the lower limit of quantification of atenolol. (A) [^3^H]sucrose after ICV administration, (B) [^14^C]sucrose after IC administration, (C) inulin after ICV administration, (D) [^14^C]inulin after IC administration, (E) morphine-6-glucuronide after ICV administration, (F) morphine after ICV administration, (G,H) atenolol after ICV administration, (I,J) acetaminophen after ICV administration, (K) antipyrine after ICV administration, (L) cefodizime after ICV administration, (M) guanidinosuccinic acid after ICV administration and (N) ziconotide after IT administration. CM: cisterna magna; ECF: brain extracellular fluid; ICV: intracerebroventricular; IT: intrathecal; M6G: morphine-6-glucuronide; SAS: subarachnoid space; Figure S2: PK profiles of morphine after ICV administration simulated with the LeiCNS-PK3.1 model plus brain–spinal cord surface compartment. (A) Model structure, (B) parameters estimated by model fitting, (C) simulated vs. observed PK profiles in cisterna magna and (D) subarachnoid space. A black syringe icon and two magnifying glass icons in (A) represent the site of administration and measurement, respectively. BCSFB: blood–cerebrospinal fluid barrier; BSC: brain–spinal cord surface compartment; CF_PPA_: correction factor for paracellular permeability; CL_BSC_: clearance from BSC to the systemic circulation; CM: cisterna magna; LV: lateral ventricles; MV: brain microvessel; Q_ex_: exchange clearance between SAS and BSC, fixed to 100 mL/min assuming fast exchange due to the proximity; SAS: subarachnoid space; sasQ_CSF,abs_: drug-independent transfer clearance from SAS to the systemic circulation by passive CSF absorption; TFV: third and fourth ventricles; V_BSC_: distribution volume of BSC; Figure S3: PK profiles of acetaminophen and antipyrine after ICV administration simulated with the LeiCNS-PK3.1 model plus periventricular clearance. (A) Model structure, (B) parameters estimated by model fitting, (C) simulated vs. observed PK profiles of acetaminophen and (D) antipyrine in cisterna magna and plasma. A black syringe icon and a magnifying glass icon in (A) represent the site of administration and measurement, respectively. BCSFB: blood–cerebrospinal fluid barrier; CF_PPA_: correction factor for paracellular permeability; CL_PV_: periventricular clearance; CM: cisterna magna; LV: lateral ventricles; MV: brain microvessel; SAS: subarachnoid space; TFV: third and fourth ventricles. References [68,69,70,71,72,73,74,75,76] are cited in Supplementary Materials.

## Abbreviations

AF: asymmetry factor; ASO: antisense oligonucleotide; BBB: blood–brain barrier; BCSFB: blood–CSF barrier; BSC: brain/spinal cord surface compartment; BSCB: blood–spinal cord barrier; CF_PPA_: correction factor for PPA; cisQ_CSF_: CSF flow rate from CM to SAS in the unidirectional CSF flow model; cisQ_CSF,D_: downward CSF movement rate from CM to SAS; CL: clearance; CM: cisterna magna; CNS: central nervous system; CP: choroid plexus; CP_MV_: choroid plexus microvessel; CSF: cerebrospinal fluid; DTPA: diethylenetriamine pentaacetate; ECF: extracellular fluid; GSA: guanidinosuccinic acid; IC: intracisternal; ICV: intracerebroventricular; IIV: interindividual variability; IT: intrathecal; IV: intravenous; Kp_uu,ECF/CSF_: the ratio of the unbound drug concentration in brain ECF or CSF to that in plasma at steady state after IV administration; LV: lateral ventricle; MV: microvessel; MW: molecular weight; PBPK: physiologically based pharmacokinetic; PPA: paracellular permeability; Q_CSF_: constant CSF flow rate in the unidirectional CSF flow model; RUV: residual unexplained variability; SAS: subarachnoid space; sasQ_CSF_: transfer CL from SAS to the systemic circulation; TFV: third and fourth ventricles; U/D ratio: the ratio between upward (caudocranial) and downward CSF movement rates, venQ_CSF,D_: downward (craniocaudal) CSF movement rate between LV, TFV and CM.
