# Supplementary material for: Revisiting Cerebrospinal Fluid Flow Direction and Rate in Physiologically Based Pharmacokinetic Model
Source: Pharmaceutics. 2022 Aug 24;14(9):1764. doi: 10.3390/pharmaceutics14091764 (PMC9504371; doi:10.3390/pharmaceutics14091764)
Supplement: Supplementary file 1 [file pharmaceutics-14-01764-s001.zip › pharmaceutics-1848443-supplementary.pdf]

pharmaceutics

Supplementary material

Article

Revisiting Cerebrospinal Fluid Flow Direction and Rate in Physiologically Based Pharmacokinetic Model

Makoto Hirasawa and Elizabeth C. M. de Lange *

Division of Systems Pharmacology and Pharmacy, Leiden Academic Center for Drug Research, Leiden University, 2333 CC Leiden, The Netherlands.; m.hirasawa@lacdr.leidenuniv.nl

* Correspondence: ecmdelange@lacdr.leidenuniv.nl; Tel.: +31-71-527-6330

Supplementary Equations. AF equations to calculate the influx and efflux asymmetry factors at the BBB and BCSFB.

$$AF_{BBB,ef} = -\frac{((Q_{CBF} + AF_{BBB,in} * CLt_{BBB} * PHF_{MV} + CLp_{BBB}) * Q_{ECF} + CLp_{BBB} * Q_{CBF}) * Kp_{uu,ECF} - (AF_{BBB,in} * CLt_{BBB} * PHF_{MV} + CLp_{BBB}) * Q_{CBF}}{CLt_{BBB} * PHF_{ECF} * Q_{CBF} * Kp_{uu,ECF}}$$

$$AF_{BBB,in} = -\frac{\left((Q_{CBF} + CLp_{BBB}) * Q_{ECF} + (AF_{BBB,ef} * CLt_{BBB} * PHF_{ECF} + CLp_{BBB}) * Q_{CBF}\right) * Kp_{uu,ECF} - CLp_{BBB} * Q_{CBF}}{CLt_{BBB} * PHF_{MV} * Q_{ECF} * Kp_{uu,ECF} - CLt_{BBB} * PHF_{MV} * Q_{CBF}}$$

$$AF_{LV,ef} = \frac{Q_{CPBF}*Kp_{uu,CM}*venQ_{CSF,U}*((sasQ_{CSF}+cisQ_{CSF,U})*venQ_{CSF,U}+cisQ_{CSF,D}*sasQ_{CSF}) - (sasQ_{CSF}+cisQ_{CSF,U})*Q_{CPBF}*Kp_{uu,LV}*venQ^2_{CSF,D}+((Q_{CPBF}*Q_{ECF}*Kp_{uu,ECF} - CLp_{BCSFBL}*Q_{CPBF}*Kp_{uu,LV}+(AF_{LV,in}*CLt_{BCSFBL}*PHF_{MV}+CLp_{BCSFBL})*(Q_{ECF}*Kp_{uu,ECF} - cisQ_{CSF,D}*Kp_{uu,CM}+*Q_{CPBF}))*sasQ_{CSF} - CLp_{BCSFBL}*Q_{CPBF}*cisQ_{CSF,U}*Kp_{uu,LV}+Q_{CPBF}*Q_{ECF}*cisQ_{CSF,U}*Kp_{uu,ECF}+(AF_{LV,in}*CLt_{BCSFBL}*PHF_{MV}+CLp_{BCSFBL})*(Q_{ECF}*cisQ_{CSF,U}*Kp_{uu,ECF}+Q_{CPBF}*cisQ_{CSF,U}))*venQ_{CSF,D}}{venQ_{CSF,D}*CLt_{BCSFBL}*PHF_{LV}*Q_{CPBF}*Kp_{uu,LV}*(sasQ_{CSF}+cisQ_{CSF,U})}$$

$$AF_{LV,in} = -\frac{(Q_{CPBF}*Kp_{uu,CM}*venQ_{CSF,U}*((sasQ_{CSF}+cisQ_{CSF,U})*venQ_{CSF,U}+cisQ_{CSF,D}*sasQ_{CSF}) - (sasQ_{CSF}+cisQ_{CSF,U})*Q_{CPBF}*Kp_{uu,LV}*venQ^2_{CSF,D}+(((Q_{CPBF}+CLp_{BCSFBL})*Q_{ECF}*Kp_{uu,ECF} - CLp_{BCSFBL}*cisQ_{CSF,D}*Kp_{uu,CM}+CLp_{BCSFBL}*Q_{CPBF})*sasQ_{CSF} - (AF_{LV,ef}*CLt_{BCSFBL}*PHF_{LV}+CLp_{BCSFBL})*Q_{CPBF}*Kp_{uu,LV}*(sasQ_{CSF}+cisQ_{CSF,U})+cisQ_{CSF,U}*((Q_{CPBF}+CLp_{BCSFBL})*Q_{ECF}*Kp_{uu,ECF}+CLp_{BCSFBL}*Q_{CPBF}))*venQ_{CSF,D}}{((Q_{ECF}*Kp_{uu,ECF} - cisQ_{CSF,D}*Kp_{uu,CM}+Q_{CPBF})*sasQ_{CSF}+Q_{ECF}*cisQ_{CSF,U}*Kp_{uu,ECF}+Q_{CPBF}*cisQ_{CSF,U})*CLt_{BCSFBL}*PHF_{MV}*venQ_{CSF,D}}$$

$$AF_{TFV,ef} = -\frac{Q_{CPBF}*Kp_{uu,CM}*venQ_{CSF,U}*((sasQ_{CSF}+cisQ_{CSF,U})*venQ_{CSF,U}+((cisQ_{CSF,D}+CLp_{BCSFBTF})*sasQ_{CSF}+CLp_{BCSFBTF}*cisQ_{CSF,U})) - (sasQ_{CSF}+cisQ_{CSF,U})*Q_{CPBF}*Kp_{uu,LV}*venQ^2_{CSF,D}+((Q_{CPBF}*cisQ_{CSF,D}*Kp_{uu,CM}+(AF_{TFV,in}*CLt_{BCSFBTF}*PHF_{MV}+CLp_{BCSFBTF})*(cisQ_{CSF,D}*Kp_{uu,CM} - Q_{ECF}*Kp_{uu,ECF} - Q_{CPBF}))*sasQ_{CSF} - (AF_{TFV,in}*CLt_{BCSFBTF}*PHF_{MV}+CLp_{BCSFBTF})*cisQ_{CSF,U}*(Q_{ECF}*Kp_{uu,ECF}+Q_{CPBF}))*venQ_{CSF,D}+CLp_{BCSFBTF}*Q_{CPBF}*cisQ_{CSF,D}*Kp_{uu,CM}*sasQ_{CSF}}{((sasQ_{CSF}+cisQ_{CSF,U})*venQ_{CSF,U}+cisQ_{CSF,D}*sasQ_{CSF})*CLt_{BCSFBTF}*PHF_{TFV}*Q_{CPBF}*Kp_{uu,CM}}$$

$$AF_{TFV,in} = \frac{Q_{CPBF}*Kp_{uu,CM}*venQ_{CSF,U}*((sasQ_{CSF}+cisQ_{CSF,U})*venQ_{CSF,U}+cisQ_{CSF,D}*sasQ_{CSF}+(AF_{TFV,ef}*CLt_{BCSFBTF}*PHF_{TFV}+CLp_{BCSFBTF})*(sasQ_{CSF}+cisQ_{CSF,U})) - (sasQ_{CSF}+cisQ_{CSF,U})*Q_{CPBF}*Kp_{uu,LV}*venQ^2_{CSF,D}+Q_{CPBF}*cisQ_{CSF,D}*Kp_{uu,CM}*sasQ_{CSF}*venQ_{CSF,D} - ((Q_{ECF}*Kp_{uu,ECF} - cisQ_{CSF,D}*Kp_{uu,CM} + Q_{CPBF})*sasQ_{CSF} + Q_{ECF}*cisQ_{CSF,U}*Kp_{uu,ECF} + Q_{CPBF}*cisQ_{CSF,U})*CLp_{BCSFBTF}*venQ_{CSF,D}+(AF_{TFV,ef}*CLt_{BCSFBTF}*PHF_{TFV}+CLp_{BCSFBTF})*Q_{CPBF}*cisQ_{CSF,D}*Kp_{uu,CM}*sasQ_{CSF}}{venQ_{CSF,D}*CLt_{BCSFBTF}*PHF_{MV}*(sasQ_{CSF}*(Q_{ECF}*Kp_{uu,ECF} - cisQ_{CSF,D}*Kp_{uu,CM}+Q_{CPBF})+Q_{ECF}*cisQ_{CSF,U}*Kp_{uu,ECF}+Q_{CPBF}*cisQ_{CSF,U})}$$

Supplementary equations notations

$AF_{BBB,ef}$: efflux asymmetry factor across the BBB; $AF_{BBB,in}$: influx asymmetry factor across the BBB; $AF_{LV,ef}$: efflux asymmetry factor across the BCSFB at LV; $AF_{LV,in}$: influx asymmetry factor across the BCSFB at LV; $AF_{TFV,ef}$: efflux asymmetry factor across the BCSFB at TFV; $AF_{TFV,in}$: influx asymmetry factor across the BCSFB at TFV; BBB: blood–brain barrier; BCSFB: blood–CSF barrier; $cisQ_{CSF,D}$: downward cisternal CSF movement rate; $cisQ_{CSF,U}$: upward cisternal CSF movement rate; CLp_{BBB}: paracellular transport clearance across the BBB; CLp_{BCSFBL}: paracellular transport clearance across the BCSFB at LV; $CLp_{BCSFBTF}$: paracellular transport clearance across the BCSFB at TFV; CLt_{BBB}: transcellular transport clearance across the BBB; CLt_{BCSFBL}: transcellular transport clearance across the BCSFB at LV; $CLt_{BCSFBTF}$: transcellular transport clearance across the BCSFB at TFV; CM: cisterna magna; CSF: cerebrospinal fluid; $Kp_{uu,CM}$: CM-to-plasma unbound drug concentration ratio at steady state; $Kp_{uu,ECF}$: brain extracellular fluid-to-plasma unbound drug concentration ratio at steady state; $Kp_{uu,LV}$: LV-to-plasma unbound drug concentration ratio at steady state; LV: lateral ventricles; PHF_{ECF}: pH factor of brain extracellular fluid; PHF_{LV}: pH factor of LV; PHF_{MV}: pH factor of brain microvessel; PHF_{TFV}: pH factor of TFV; Q_{CBF}: cerebral blood flow rate; Q_{CPBF}: choroid plexus blood flow rate; Q_{ECF}: brain extracellular fluid bulk flow rate; $sasQ_{CSF}$: clearance from subarachnoid space to plasma; TFV: third and fourth ventricles; $venQ_{CSF,D}$: downward ventricular CSF movement rate; $venQ_{CSF,U}$: upward ventricular CSF movement rate.

Table S1. Unbound plasma PK parameters used as input to the LeiCNS-PK models.

Molecule	Plasma PK parameter estimates						Interindividual variability				Residual unexplained variability		Reference
	CL_{cen} (mL/min)	$CL_{cen-per1}$ (mL/min)	$CL_{cen-per2}$ (mL/min)	V_{cen} (mL)	V_{per1} (mL)	V_{per2} (mL)	CL_{cen} (%)	$CL_{cen-per1}$ (%)	V_{cen} (%)	V_{per2} (%)	Proportional (%)	Additive (ng/mL)	
Sucrose	0.00387	-	-	9.2	-	-	-	-	-	-	6.52	-	[19]
Inulin	0.00463	-	-	9.6	-	-	-	-	-	-	5.79	-	[19]
Morphine-6-glucuronide	8.05	5.96	0.12	70.6	70.0	21.2	-	-	-	-	-	-	[68]
Morphine	23.3	4.97	31.7	176	1640	476	46.9	93.9	92.5	49.2	24.2	-	[6]
Atenolol	6.30	4.25	-	119	203	-	8.92	-	-	-	14.7	-	[6]
	12.1	7.40	6.67	304	466	50.5	-	-	-	-	-	0.680	[21]
Acetaminophen	0.075	11.6	-	218	4070	-	-	-	-	-	5.08	-	[21]
	14.0	3.26	-	225	86.3	-	8.14	-	6.68	-	7.67	3.80	[22]
Antipyrine	2.59	-	-	237	-	-	-	-	-	-	-	102	[21]
Cefodizime	1.49	4.85	15.1	137	151	53.4	-	-	-	-	0.93	-	[23]
Guanidinosuccinic acid	1.02	2.29	-	34.3	26.0	-	-	-	-	-	-	0.568	[24]
Ziconotide	0.0845	0.461	-	28.2	29.0	-	-	-	-	-	3.42	-	[25]

CL_{cen}: clearance from the central compartment; $CL_{cen-per1/2}$: distribution clearance between the central and peripheral compartments; V_{cen}: central distribution volume; $V_{per1/2}$: peripheral distribution volume.

Table S2. Drug-specific physicochemical parameters, unbound fraction in plasma and Kp_{uu} values.

Molecule	Molecular weight	Charge class	pK_a	pK_b	$logP_{o/w}$	fu_p	Kp_{uu} after IV administration		
							Brain ECF	CSF_{LV}	CSF_{CM}
Sucrose	342.3	Neutral	11.84	-3	-3.7	1[34]	No active transport[34]		
Inulin	6179.4	Neutral	11.27	-4	-62[a]	1[b]	No active transport		
Morphine-6-glucuronide	285.3	Base	10.26	9.12	0.87	0.83[6]	0.37[6,69]	0.20[c]	0.20[69]
Morphine	461.5	Zwitterion	2.87	9.12	0.13[d]	0.84[68-70]	0.36[68-70]	0.029[c]	0.029[69]
Atenolol	266.34	Base	14.08	9.67	0.16	0.91[6]	0.042[22]	0.51[c]	0.51[21]
Acetaminophen	151.16	Neutral	9.46	-4.4	0.91	0.81[6]	0.22[22]	1.05[c]	1.05[21]
Antipyrine	188.23	Neutral	NA	0.49	0.38	1[71]	0.71[4]	1.51[c]	1.51[21]
Cefodizime	584.67	Acid	2.68	3.45	1.07[d]	0.14[23]	0.19[23]	0.027[c]	0.027[23]
Guanidinosuccinic acid	175.14	Zwitterion	2.98	12.2	-2.07	1[72,73]	0.25[c]	0.25[c]	0.25[74],[e]
Ziconotide	2639.1	Base	3.41	12.18	-23[a]	0.44[75]	0.018[c]	0.018[c]	0.018[25]

CM: cisterna manga; CSF: cerebrospinal fluid; ECF: extracellular fluid; fu_p: fraction unbound in plasma; IV: intravenous; Kp_{uu}: the ratio of the unbound drug concentration in the compartment of interest to that in plasma at steady state; LV: lateral ventricle; NA: not applicable; pK_a: the ionization constant of the strongest acidic group; pK_b: the ionization constant of the strongest basic group.

[a] Calculated by ChemAxon [33]; [b] obtained from DrugBank [31]; [c] the same value as CSF_{CM} was assumed; [d] calculated by ALOGP [76]; [e] a mean value in human at lumbar CSF was used.

Table S3. Assumed volumes of dosing solution reached each compartment at the end of administration.

Molecule	Reference	Dosing site	Dosing volume (μL)	Assumed volume of dosing solution reached in each compartment at the end of administration (μL)				
				Left LV	Right LV	TFV	CM	SAS
[^{3}H]sucrose	[18]	Left LV	12.5[a]	3.75	0	7.5	1.25	0
[^{14}C]sucrose	[19]	CM	100[b]	0	0	0	17	83
Inulin	[22]	Right LV	12.5[c]	0	3.75	7.5	1.25	0
[^{14}C]inulin	[19]	CM	100[b]	0	0	0	17	83
Morphine-6-glucuronide	[18]	Left LV	12.5[a]	3.75	0	7.5	1.25	0
Morphine	[18]	Left LV	12.5[a]	3.75	0	7.5	1.25	0
Atenolol	[21]	Left LV[d]	10	3.75	0	6.25	0	0
	[22]	Left LV	15	3.75	0	7.5	3.75	0
Acetaminophen	[21]	Left LV[d]	10	3.75	0	6.25	0	0
	[22]	Left LV	10	3.75	0	6.25	0	0
Antipyrine	[21]	Left LV[d]	10	3.75	0	6.25	0	0
Cefodizime	[23]	Left LV[d]	10	3.75	0	6.25	0	0
Guanidinosuccinic acid	[24]	Left LV	10	3.75	0	6.25	0	0
Ziconotide	[25]	SAS	100	0	0	0	0	100

CM: cisterna manga; LV: lateral ventricle; SAS: subarachnoid space; TFV: third and fourth ventricles.

[a] Assumed as the middle of other ICV cases (10-15 μL); [b] the same volume of CSF was withdrawn before administration; [c] 50 μL/kg in the literature; [d] assumed because the site of administration was "lateral ventricle" in the literature.

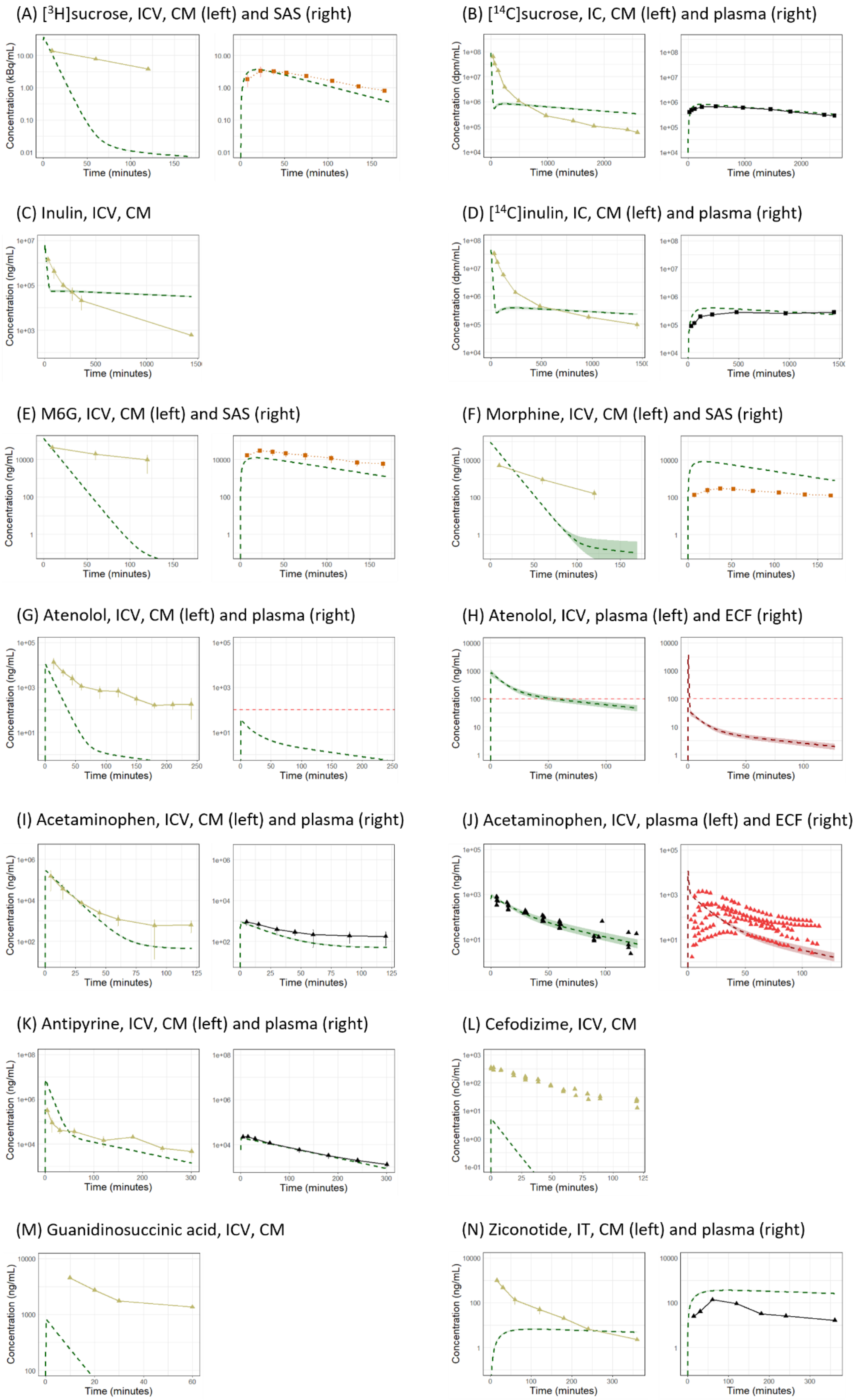

Figure S1. Comparison of simulated PK profiles of 10 molecules using the original LeiCNS-PK3.0 model with the default settings to the observed ones after intra-CSF administration. Dots and solid lines represent mean concentrations ± standard deviation or standard error in CM (khaki), SAS (orange), plasma (black) and ECF (red). Dashed lines represent the median of 200 model simulations with 95% prediction intervals (colored band). Red horizontal dashed lines in (G)

and (H) represent the lower limit of quantification of atenolol. (A) [^{3}H]sucrose after ICV administration, (B) [^{14}C]sucrose after IC administration, (C) inulin after ICV administration, (D) [^{14}C]inulin after IC administration, (E) morphine-6-glucuronide after ICV administration, (F) morphine after ICV administration, (G)(H) atenolol after ICV administration, (I)(J) acetaminophen after ICV administration, (K) antipyrine after ICV administration, (L) cefodizime after ICV administration, (M) guanidinosuccinic acid after ICV administration and (N) ziconotide after IT administration. CM: cisterna magna; ECF: brain extracellular fluid; ICV: intracerebroventricular; IT: intrathecal; M6G: morphine-6-glucuronide; SAS: subarachnoid space.

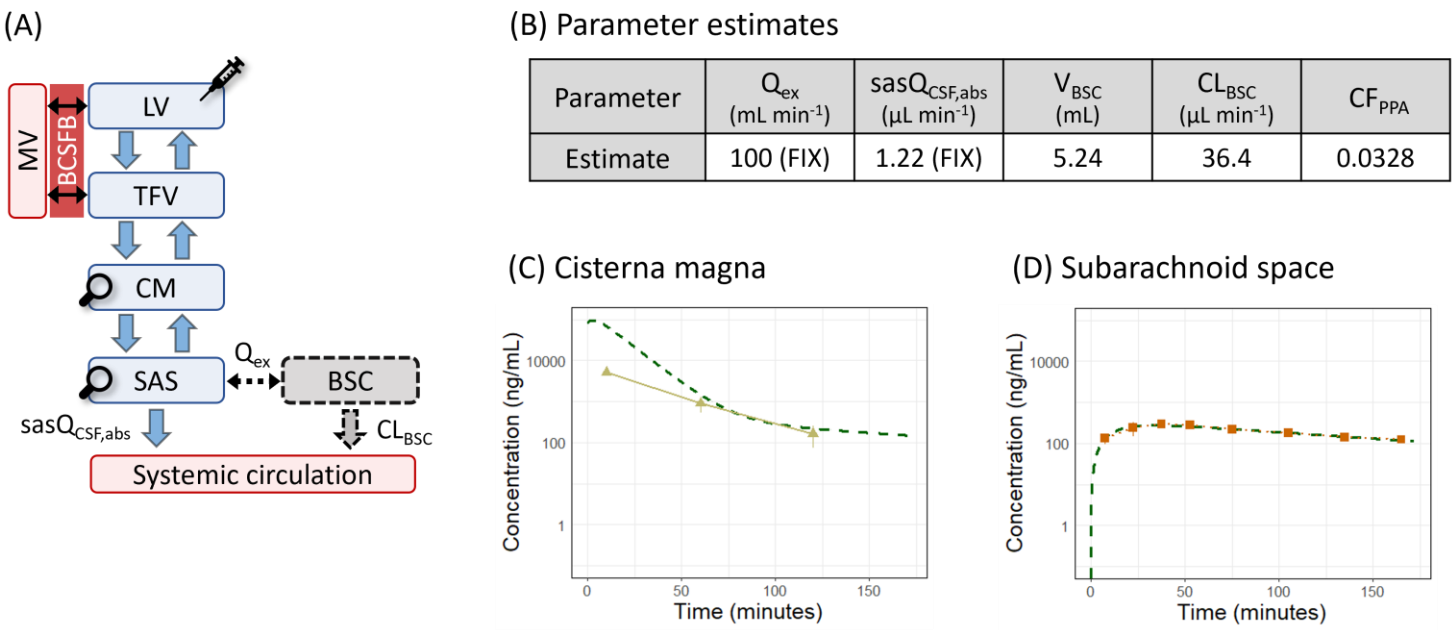

Parameter	Q_{ex} (mL min^{-1})	$sasQ_{CSF,abs}$ (μL min^{-1})	V_{BSC} (mL)	CL_{BSC} (μL min^{-1})	CF_{PPA}
Estimate	100 (FIX)	1.22 (FIX)	5.24	36.4	0.0328

Figure S2. PK profiles of morphine after ICV administration simulated with the LeiCNS-PK3.1 model plus brain–spinal cord surface compartment. (A) Model structure, (B) parameters estimated by model fitting, (C) simulated vs. observed PK profiles in cisterna magna and (D) subarachnoid space. A black syringe icon and two magnifying glass icons in (A) represent the site of administration and measurement, respectively. BCSFB: blood–cerebrospinal fluid barrier; BSC: brain–spinal cord surface compartment; CF_{PPA}: correction factor for paracellular permeability; CL_{BSC}: clearance from BSC to the systemic circulation; CM: cisterna magna; LV: lateral ventricles; MV: brain microvessel; Q_{ex}: exchange clearance between SAS and BSC, fixed to 100 mL/min assuming fast exchange due to the proximity; SAS: subarachnoid space; $sasQ_{CSF,abs}$: drug-independent transfer clearance from SAS to the systemic circulation by passive CSF absorption; TFV: third and fourth ventricles; V_{BSC}: distribution volume of BSC.

(A)

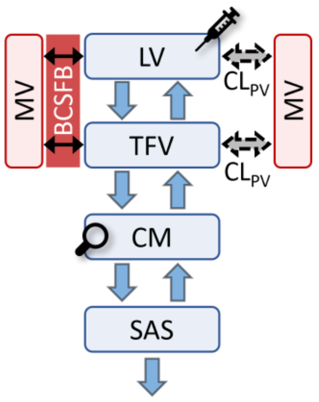

(B) Parameter estimates[a)]

Parameter	sasQ_{CSF} (μL min^{-1})	CL_{PV} (μL min^{-1})
Acetaminophen	5.17	21.7
Antipyrine	0.632	148

[a)] CF_{PPA} was set to 1 for parameter estimation

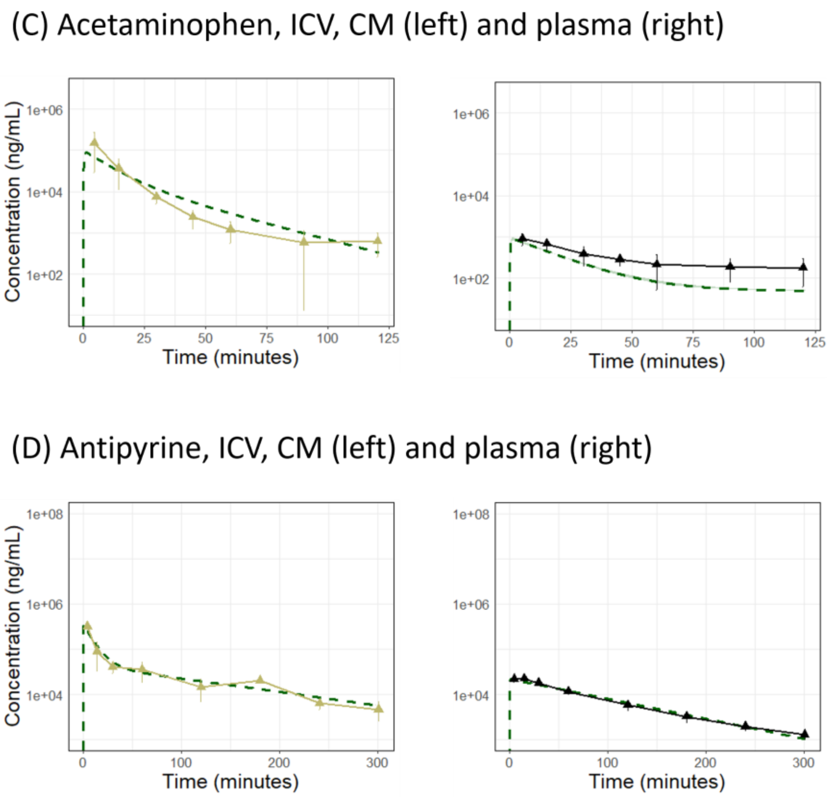

Figure S3. PK profiles of acetaminophen and antipyrine after ICV administration simulated with the LeiCNS-PK3.1 model plus periventricular clearance. (A) Model structure, (B) parameters estimated by model fitting, (C) simulated vs. observed PK profiles of acetaminophen and (D) antipyrine in cisterna magna and plasma. A black syringe icon and a magnifying glass icon in (A) represent the site of administration and measurement, respectively. BCSFB: blood–cerebrospinal fluid barrier; CF_{PPA}: correction factor for paracellular permeability; CL_{PV}: periventricular clearance; CM: cisterna magna; LV: lateral ventricles; MV: brain microvessel; SAS: subarachnoid space; TFV: third and fourth ventricles.
